# Effects of Biomimetic Shoes on Healthy Young Children's Gait

**DOI:** 10.1055/s-0043-1771006

**Published:** 2023-10-24

**Authors:** Liria Akie Okai-Nobrega, Thiago Ribeiro Teles Santos, Ana Paula Lage, Priscila Albuquerque de Araújo, Thales Rezende Souza, Sergio Teixeira Fonseca

**Affiliations:** 1 Departamento de Fisioterapia, Universidade Federal de Minas Gerais, Belo Horizonte, MG, Brasil; 2 Anamê Ciência e Tecnologia em Produtos para Saúde Infantil, Belo Horizonte, MG, Brasil; 3 Faculdade de Educação Física e Fisioterapia, Universidade Federal de Uberlândia, Uberlândia, MG, Brasil

**Keywords:** biomechanics, biomimetics, child, gait, infant, shoes, walking

## Abstract

**Objective** To compare the spatial-temporal parameters and walking kinematics of toddlers wearing biomimetic shoes, regular shoes (daily use owned shoes), and barefoot.

**Methods** Spatial-temporal parameters (speed, step length, and stride width), the mean vertical displacement of the center of mass (COM), knee flexion peak, and maximal foot height were analyzed.

**Results** Children were not different in biomimetic shoes and barefoot conditions on speed, step length, and COM vertical displacement. There was no difference among conditions on stride width and foot height. The knee flexion peak was greater in shod conditions than barefoot. The regular shoes showed greater COM vertical displacement than biomimetic shoes and barefoot.

**Conclusion** The findings showed that shoes affected the walking pattern in young children, but a shoe with a biomimetic design had a lesser effect on the walking pattern.

## Introduction

Shoes are the primary interface between the body and the ground. They contribute to how ground reaction forces are applied to the foot and transferred to the entire body. 1 2 The effect of shoes on young children's gait is poorly understood. 3 The walking alone developmental landmark happens in toddlers, on average, around twelve months. 4 It is a new experience since they almost had never practiced the dynamic bipedal and unipedal stances necessary to master walking. 5 Furthermore, the gait pattern adopted by young children differs from adults due to anthropometrics (e.g., body mass distribution) and continuous refinements characteristic of task development. 6 The unique characteristics of young children's gait contribute to the importance of external elements, such as shoes, in developing this critical motor benchmark. 7

Clothing, footwear, and different terrains influence children's movements but are rarely reported in research studies. 8 Urban life establishes mandatory requirements for shoes to comply with social and safety rules. The primary role of shoes is to protect the toddler's foot from injury due to rough or uneven surfaces, excessive impact, and cold and wet environments. 8 9 10 Shoes provide many benefits but also have disadvantages 11 It has been suggested that optimum foot development occurs in the barefoot environment. 8 9 10 Therefore, children's shoe designs should provide an experience similar to the barefoot condition, considering shock absorption and load distribution. 10 12

Walking barefoot develops foot muscle strength and mobility and contributes to the variability in the medial plantar arch. 9 13 Moving around in nature often involves walking on a soft substratum such as sand, affecting locomotion mechanics and energy. 14 Walking in the real world involves negotiating challenges over uneven surfaces, requiring constant adjustments of the body's movement pattern to maintain stability. Michael et al. 15 stated that anatomically shaped shoes permit biologically normal structure and foot function. Taking inspiration from a natural design (i.e., biomimetism), a commercially available shoe for children at a young age used sand as a model to develop its midsole through polymers to simulate walking over a soft substratum. As a nature-inspired design, this shoe claims to respect the morphology of the toddler's feet (rounded forefoot) and provide better conditions for foot development. 16

This study investigated whether using biomimetic shoes affects young children's walking patterns. More specifically, this study aimed to compare the spatial-temporal parameters and walking kinematics of toddlers wearing biomimetic shoes, regular shoes (daily use owned shoes), and barefoot. This work hypothesized that walking with biomimetic shoes would have a similar pattern to the barefoot condition than regular shoes.

## Methods

### Study Design and Participants

This experimental study was a cross-sectional, repeated measures trial. Twenty typically developing children (1 to 3 years old) participated in this study. The sample was recruited based on a convenience sampling method with the help of the universitýs students and local community members. The inclusion criteria were children full term of birth, typically developed without prior history of significant medical issues, capable of independent walking (i.e., walking without support), and foot size between 10 to 16.6 cm. The exclusion criteria were children over 3 years old, complaining of any pain during gait *,* using any systematic neurologic medication, and unable to complete the entire measurement setup. Informed consent was obtained from all the parents, and the procedures were approved by the University's Ethics Review Committee (66806317.1.0000.5149).

### Procedures

**Fig. 1:**
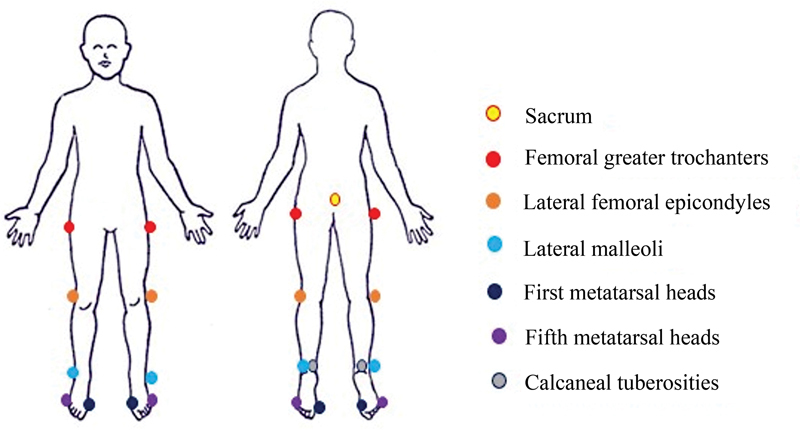
Marker setup protocol used to collect spatial-temporal and kinematic parameters.

**Fig. 2:**
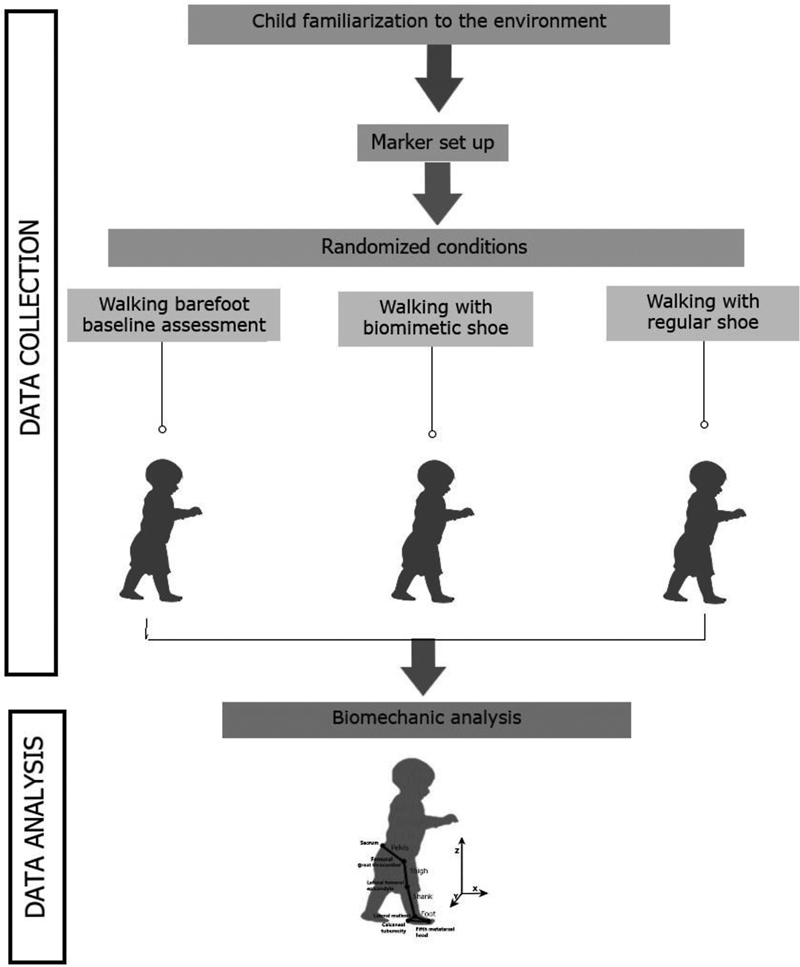
Flowchart showing from data collection to data analysis: i) child was familiarized with the biomechanical collection environment; ii) passive markers protocol was applied to anatomical landmarkers; iii) child was oriented to walk in three randomized conditions (barefoot, biomimetic shoes and regular shoes); iv) biomechanical analysis.

**Fig. 3:**
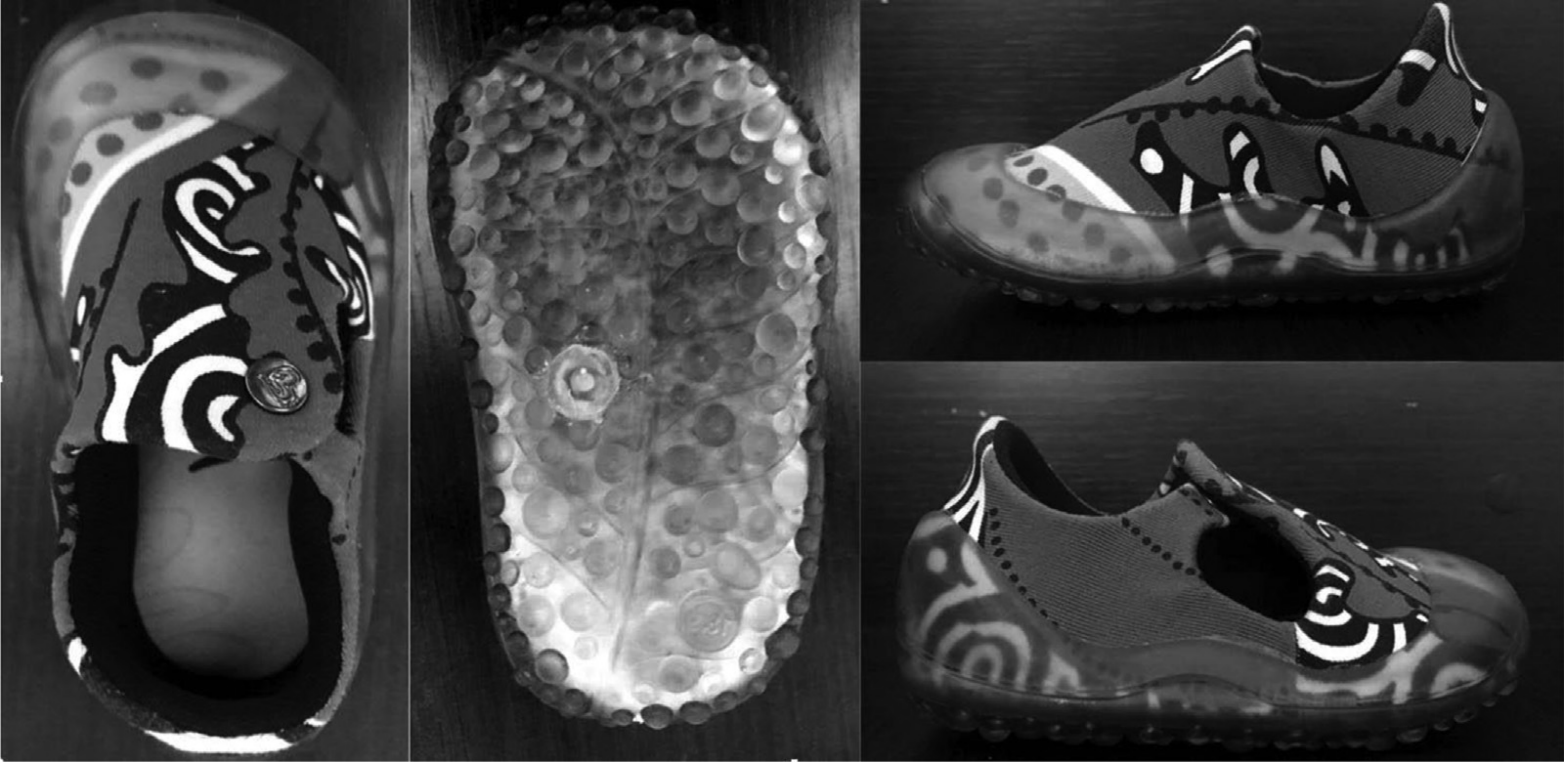
The biomimetic shoe. Images from left to right show the shoe's up, down, medial side, and lateral side views.

**Fig. 4:**
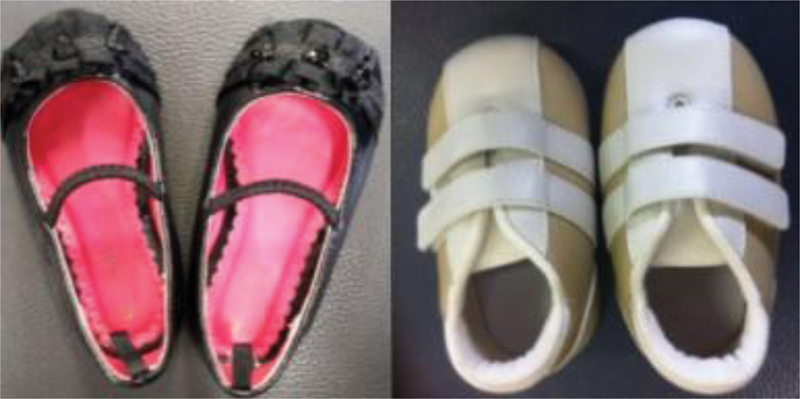
Image with two exemplars of daily used regular shoes.

The laboratory settings and the experimental procedures were adapted to offer a comfortable environment to the children. Before gait recording, one parent initially held the child by the hand to explore the environment and get used to it. Lower limb kinematic data were acquired based on 13 retro-reflective markers (diameter of 8 mm) placed on the sacrum, femoral greater trochanters, lateral femoral epicondyles, lateral malleoli, calcaneal tuberosities, first and fifth metatarsal heads ( Fig. 1 ). Bilateral lower limb kinematics were collected to consider functional asymmetries described in previous studies in healthy toddlers. 5 Mainly lateral markers were used since pilot tests showed that the medial markers frequently fall, thus needing re-attachment during the assessment. The data were collected during gait using Qualisys ProReflex MCU motion analysis system (QUALISYS MEDICAL AB®, Gothenburg, Switzerland) at a sample rate of 120 Hz. A flowchart showing from data collection to the kinematic analysis is shown in Fig. 2 . Children walked in three randomized testing conditions: barefoot, biomimetic shoe ( Fig. 3 – Noeh®, www.noeh.com.br , Noeh Baby, Brazil), and regular shoe (daily use owned footwear – Fig. 4 ). The barefoot condition was collected as the baseline assessment of gait parameters to compare with the shod conditions. The biomimetic shoe ( Fig. 3 ) presents a biomimetic design with a dynamic midsole, as described by Lage (2014). Besides, no children were familiarized with this biomimetic shoe before data collection. The shoe size was checked a priori to ensure the appropriate fit. For the regular shoes ( Fig. 4 ), the parents were asked to bring the most comfortable and daily used covered shoes apart from flip flops or open sandals.All children wore only disposable diapers for the data acquisition. Each participant walked around ten trials in each experimental condition.

During data recording, the children were encouraged to walk toward a parent or researcher at a self-selected speed. Children walked over a raised platform (10 cm high), and all procedures were performed by the same experienced examiner and three trained scientific assistants. A person responsible for the child and one research staff remained alongside the children to prevent them from falling.

### Data Analysis

**Fig. 5:**
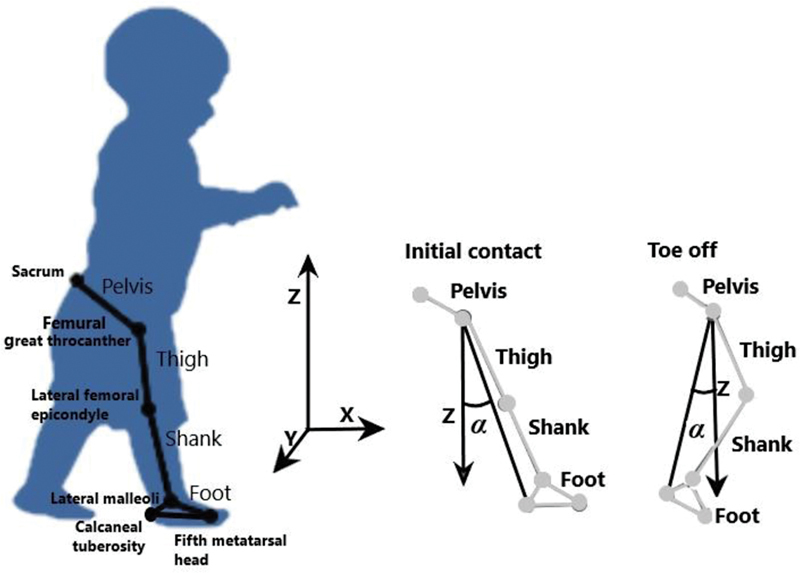
Anatomical markers used to model lower limb body segments: pelvis, thigh, shank, and foot. Elevation angles (α) were computed relative main limb axis (from femoral great trochanter to calcaneal tuberosity) and vertical (Z). Initial contact was determined as the highest elevation angle (α) and foot-off as the lowest elevation angle (α).

Data files were tracked using Qualisys Track Manager Software (QUALISYS MEDICAL AB®, Gothenburg, Switzerland). Kinematic data were exported to be processed under Visual3D software (C-Motion, Inc., Rockville, USA). Raw data were initially filtered using a lowpass, fourth-order Butterworth filter with a cut-off of 6 Hz. The following limb segments were defined as stick figures ( Fig. 5 ): pelvis (a line connecting from the sacrum marker to the femoral greater trochanter marker), thigh (a line connecting from femoral great trochanter marker to the lateral femoral epicondyle marker), shank (a line connecting from lateral femoral epicondyle to lateral malleolus marker), and foot (a line connecting from calcaneal tuberosity marker to the medium point between first and fifth metatarsal heads). The events necessary to define the gait cycle were created based on the method adapted for toddlers by Ivanenko et al. 17 Briefly, the elevation angle (α in Fig. 5 ) of each limb corresponds to the angle between the main limb axis and the vertical (z). Initial contact and foot-off were determined with the higher and lower elevation angle values, respectively. Furthermore, all events were visually checked individually, and adjustments were made when necessary.

Thirty to forty gait cycles from each subject were chosen for analysis. Each gait cycle was normalized to 100% (initial contact to the following initial contact). The following spatial-temporal parameters were extracted: walking speed, step length, and stride width. Also, the mean vertical displacement of the body's center of mass (COM) 18 was calculated based on the retro-reflective marker placed on the sacrum. Studies showed that this marker could essentially estimate the vertical displacement of the COM. 18 Finally, the following variables related to the elevation of the foot from the ground were calculated: the knee flexion peak 19 20 and maximal foot height during the swing phase. The maximal foot height was considered the maximum peak of vertical displacement of the foot́s center of mass.

### Statistical Analysis

The assumptions of normality and homogeneity of variance were confirmed before running the inferential tests. A one-way repeated-measures analysis of variance (ANOVA) was used to investigate the effect of condition (barefoot, biomimetic shoes, and regular shoes) on speed, stride width, and mean vertical displacement of COM. A two-way repeated-measures ANOVA was used to investigate the effect of the condition and the side (right and left) on step length, knee flexion peak, and maximal foot height. A *post hoc* test was performed to identify pairwise differences when the ANOVA identified a significant main effect. A type I error probability of 0.05 was considered for all the analyses. Cohen's *f* and *dz* effect size was calculated and interpreted as follows: small ( *f* = 0.10 and *dz* = 0.20), medium ( *f* = 0.25 and *dz* = 0.50), and large ( *f* = 0.40 and *dz* = 0.80) (Cohen, 1969). Statistical analysis was performed using IBM SPSS Statistics 22.0 (IBM Corp., Armonk, NY, USA).

## Results

Table 1 shows descriptive data, p-values, effect sizes, and statistical power of the inferential statistics for spatial-temporal and kinematics parameters. Initially, twenty children were recruited; however, nineteen children's data (52% of girls, age: 14.8 ± 2.0 months, body mass: 10.23 ± 1.19 kg, and height: 0.77 ± 0.04 m) were analyzed. They all had more than one month of walking alone experience. One toddler was excluded since the child refused to perform one of the investigated conditions. Besides, one child presented knee angle data of only one side at the regular shoe condition due to missing data on the other side. All children came with a regular, daily use shoe: all flat shoes (no heel height): 33.3% wearing shoes, as illustrated in Fig. 4 (right), and 66,6% with shoes, as shown in Fig. 4 (left).

**Table 1:** Descriptive and inferential statistics of spatiotemporal and kinematics variables ( *n* = 19)

Variable	Descriptive statistics – Mean (SD)						ANOVA			*Post hoc –* Condition Main Effect		
	Biomimetic shoe		Barefoot		Regular shoe		Main effect		Interaction effects	Biomimetic shoe X Barefoot	Biomimetic shoe X Regular shoe	Barefoot X Regular shoe
	Right	Left	Right	Left	Right	Left	Condition	Side				
*Spatial-temporal parameters*												
Speed ( *m/s* )	0.59 (0.20)		0.63 (0.21)		0.69 (0.24)		*p* = 0.05* F (2,36) = 3.21 *f* = 0.42 Power = 0.58			*p* = 0.28 *t* (18) = -1.12 *dz* = 0.26 Power = 0.18	*p* = 0.02* *t* (18) = -2.52 *dz* = 0.58 Power = 0.66	*p* = 0.19 *t* (18) = -1.37 *dz* = 0.31 Power = 0.25
Step length ( *m* )	0.23 (0.05)	0.21 (0.06)	0.22 (0.05)	0.22 (0.05)	0.24 (0.06)	0.24 (0.06)	*p* = 0.04* F (2,36) = 3.58 *f* = 0.45 Power = 0.63	*p* = 0.35 F (1,18) = 0.92 *f* = 0.23 Power = 0.15	*p* = 0.06 F (2,36) = 3.08 *f* = 0.42 Power = 0.56	*p* = 0.96 *t* (18) = -0.06 *dz* = 0.01 Power = 0.05	*p* = 0.02* *t* (18) = -2.49 *dz* = 0.57 Power = 0.65	*p* = 0.06 *t* (18) = -1.98 *dz* = 0.45 Power = 0.47
Stride width ( *m* )	0.11 (0.03)		0.11 (0.02)		0.11 (0.02)		*p* = 0.71 F (2,36) = 0.34 *f* = 0.14 Power = 0.10					
*Kinematic parameters*												
COM mean vertical displacement ( *m* )	0.016 (0.006)		0.016 (0.006)		0.019 (0.006)		*p* = 0.02* F (2,36) = 4.13 *f* = 0.48 Power = 0.69			*p* = 0.82 *t* (18) = -0.23 *dz* = 0.05 Power = 0.06	*p* = 0.01* *t* (18) = -2.99 *dz* = 0.68 Power = 0.81	*p* = 0.05* *t* (18) = -2.06 *dz* = 0.47 Power = 0.50
Knee flexion peak (°)	76.5 (9.4)	71.1 (9.0)	71.0 (11.0)	67.5 (9.2)	75.0 (9.1)	75.1 (13.8)	*p* = 0.01* F (1.5,34) = 6.48 *f* = 0.62 Power = 0.80	*p* = 0.06 F (1,17) = 3.92 *f* = 0.48 Power = 0.46	*p* = 0.19 F (1.3,34) = 1.73 *f* = 0.31 Power = 0.27	*p* < 0.01* *t* (18) = 3.92 *dz* = 0.90 Power = 0.96	*p* = 0.48 *t* (17) = -0.73 *dz* = 0.17 Power = 0.10	*p* = 0.01* *t* (17) = -2.81 *dz* = 0.66 Power = 0.73
Maximal foot height ( *m* )	0.098 (0.017)	0.096 (0.017)	0.089 (0.014)	0.089 (0.013)	0.093 (0.019)	0.092 (0.018)	*p* = 0.07 F (2,36) = 2.85 *f* = 0.40 Power = 0.52	*p* = 0.41 F (1,18) = 0.72 *f* = 0.20 Power = 0.13	*p* = 0.87 F (2,36) = 0.15 *f* = 0.10 Power = 0.07			

**Note:** SD = Standard deviation; COM = Center of mass, ^*^ = *p* £ 0.05; *F* (x,y) = F statistic (degrees of freedom), *f* = Cohen's f effect size, *dz* = Cohen's *dz* effect size.

### Spatial-temporal Parameters

Speed was different among conditions ( Table 1 ). *Post hoc* analysis showed that children walked slower when using biomimetic shoes than regular shoes. Biomimetic and regular shoe conditions were not different from barefoot.

Step length was also different among conditions ( Table 1 ). *Post hoc* analysis showed that children presented a shorter step length using biomimetic shoes than regular shoes. There was no difference in step length between shod conditions and barefoot. Also, there was no difference between right and left step lengths and interaction effect between condition and side.

Stride width was not different among conditions ( Table 1 ).

### Kinematic Parameters

The mean vertical displacement of COM was greater in the regular shoe condition than in barefoot and biomimetic shoe conditions ( Table 1 ). Besides, this variable did not present a statistical difference between biomimetic shoe and barefoot conditions.

Knee flexion peak was also different among conditions. *Post hoc* analysis showed that shod conditions had a greater knee flexion peak than barefoot. This variable was not different between shod conditions. Also, there was no difference between right and left knee flexion peaks and interaction effect between condition and side.

The maximal foot height during the swing phase was not different among conditions, between sides, and there was no interaction between condition and side.

## Discussion

The major finding of this study was that biomimetic-designed shoes have a smaller impact than regular shoes on spatial-temporal parameters and walking kinematics in healthy young children. This study compared the walking characteristics of young children in three conditions: biomimetic shoes, daily used regular shoes, and barefoot. There is limited and fair quality evidence that children's footwear alters biomechanical gait parameters. 20 21 The current work indicated that biomimetic-designed shoes have a minor impact on younger children's walking patterns, and it is similar to barefoot walking.

As a global walking measure, spatial-temporal parameters can provide insight into the differences among the three walking conditions. Significant differences with a large effect size were found among conditions in walking speed and step length. Previous studies reported that shod walking results in increased walking speed compared to barefoot. 2 22 On the other hand, others did not find this difference. 3 7 20 23 In the current study, the gait speed with the biomimetic shoe was not different from barefoot; however, the biomimetic condition differed from the regular shoe condition. The biomimetic midsole provides an irregular contact surface to the plantar foot region. This challenge may have contributed to reducing children's gait speed. Shkuratova et al. 24 described that slower walking speed might improve walking stability. When walking speed was directly manipulated in younger adults, slower speeds decreased local dynamic instability, despite increased variability. 24 Thus, reducing velocity could be an adaptation for the children to improve walking stability when using non-usual shoes that provide a softer contact surface for their feet.

Step length was longer in regular shoes compared with biomimetic shoes.The longer step length when wearing regular shoes agrees with the previous studies. 2 10 20 22 23 However, using a biomimetic shoe was not different from being barefoot. Furthermore, the mean vertical displacement of COM was also not affected by the biomimetic shoe compared to barefoot. The COM motion during gait can represent a summary indicator of the movement of the whole-body system. 25 26 This behavior can provide general information about the mechanics of walking concerning energy expenditure and mechanical efficiency. Our results indicated that the mean vertical displacement of COM with regular shoes was greater than the other two conditions. This result could be related to the higher velocity 26 27 in regular shoe condition, also presented in our data. Besides, this COM behavior difference may contribute to a higher metabolic cost of walking 28 with regular shoes than with biomimetic shoes and barefoot.

The knee flexion peak differed between shod conditions and barefoot (large effect size). Our results follow the literature 2 since a slight increase in the knee flexion peak was observed in the shod conditions compared to the barefoot condition. This change could be attributed to the added weight related to the shoes. Increased weight of shoes distally may increase knee flexion during the gait. Dominici et al. 29 concluded that a higher foot lift could be a safe, simple strategy for avoiding potential stumbling and falls and reducing the effect of involuntary foot drag and reduced dorsiflexion activity.

To our knowledge, no study investigated the impact of biomimetic-designed footwear on children. The current study did not track body segments three-dimensionally, limiting the results. Three-dimensional tracking requires more retro-reflective markers attached to children. During the pilot study, we found that some children do not tolerate multiple markers attached to their bodies. Besides, the anatomical markers used to define foot segment were placed over the shoes and not directly on the foot skin. Another limitation was that the participants used different regular shoes. Since they chose the most usual and comfortable, the characteristics of regular shoes differed across all participants. Moreover, the findings of this study were the result of short-term use. Thus, our research did not consider biomimetic shoes' impact on long-term management. Further research on biomimetic shoe design wearing for long periods could enhance understanding of this footwear effect.

## Conclusion

The present study showed that biomimetic shoes' use resulted in speed, step length, stride width, and COM vertical displacement not different from barefoot walking in young children during gait onset. The knee flexion peak was affected by the biomimetic shoes like the regular shoes. The regular shoes resulted in greater COM vertical displacement than biomimetic shoes and barefoot. Therefore, the biomimetic shoe design may provide a walking experience similar to barefoot, with less impact on the walking pattern.
