# Supplementary material for: Effects of Biomimetic Shoes on Healthy Young Children's Gait
Source: Rev Bras Ortop (Sao Paulo). 2023 Oct 24;59(3):e435–42. doi: 10.1055/s-0043-1771006 (PMC11214844; doi:10.1055/s-0043-1771006)
Supplement: Related texts

# Research article: Efeitos do uso de calçado biomimético na marcha de crianças típicas

**Keywords:** biomecânica, biomimético, caminhada, criança, lactente, marcha, sapatos

## Abstract

**Objetivo** O objetivo deste trabalho foi comparar os parâmetros espaço temporais e cinemáticos da marcha de crianças típicas em três condições: descalças, usando calçados biomiméticos e, calçados de uso diário (não biomiméticos - próprios das crianças).

**Métodos** Foram analisadas variáveis espaço temporais (velocidade, comprimento e largura da passada), deslocamento vertical do centro de massa (CM), pico de flexão do joelho e altura máxima do pé, coletados via avaliação tridimensional do movimento.

**Resultados** Comparado com a condição descalça, o uso do calçado biomimético não foi estatisticamente diferente em relação a velocidade da marcha, comprimento da passada e altura do pé. A largura da passada e a altura do pé não foi diferente estatisticamente entre as condições estudadas. O pico de flexão do joelho foi maior nas condições com calçados comparado a condição descalça. Os calçados de uso diário apresentaram maior deslocamento vertical do COM do que nas condições com o calçado biomimético e descalço.

**Conclusão** Os achados deste trabalho reafirmam que o uso do calçado influencia a marcha de crianças, especificamente na fase de desenvolvimento da marcha mas, que calçados com um design biomimético tem menores impactos no padrão de marcha das crianças.

## Introdução

Calçados são a interface primária entre o corpo e o solo e contribuem na forma em que as forças de reação do solo são aplicadas ao pé e transferidas para o corpo todo. 1 2 Poucos são os estudos que avaliam os efeitos dos calçados na marcha em crianças na fase de desenvolvimento e aquisição da marcha. 3 A marcha independente é um marco do desenvolvimento motor que acontece em bebês, em média, por volta dos doze meses de idade. 4 Nessa fase, a marcha é uma experiência nova para essas crianças, uma vez que nunca haviam experienciado concomitantemente, a postura ortostáticas associadas as posturas dinâmicas bipodais e unipodais necessárias para o domínio da marcha. 5 Além disso, o padrão de marcha adotado por crianças pequenas difere dos adultos devido à sua própria antropometria (por exemplo, distribuição de massa corporal) e refinamentos contínuos característicos do desenvolvimento de tarefas motoras. 6 Essas características específicas da marcha de crianças pequenas contribuem para a importância de elementos externos, como calçados, que interferem diretamente, no desenvolvimento desse marco motor crítico. 7

Detalhes experimentais como roupas, sapatos e diferentes tipos de solos aos quais as crianças caminham, influenciam os movimentos das crianças, mas raramente são relatados em pesquisas. 8 Apesar disso, a vida urbana impõe regras sociais e de segurança como o uso de calçados. O principal papel dos calçados é proteger os pés das crianças de lesões decorrentes de superfícies ásperas ou irregulares, impacto excessivo e ambientes frios e úmidos. 8 9 10 Apesar dos seus muitos benefícios, os calçados trazem também desvantagens. Há muitos estudos que indicam que o desenvolvimento ideal do pé ocorre em condições sem o uso do sapato, ou seja, descalços. 8 9 10 Dessa forma, os *designs* de sapatos infantis deveriam proporcionar uma experiência semelhante à condição de pés descalços, considerando a absorção de choque e a distribuição de carga. 10 12

O andar descalço desenvolve a força muscular e a mobilidade do pé e contribui para uma maior variabilidade de condições e estímulos no arco plantar medial. 9 13 A movimentação descalça em solos naturais permite a marcha sobre um substrato macio, como areia, o que afeta a mecânica e a energia de locomoção. 14 O caminhar no mundo real requer enfrentar desafios em superfícies irregulares, o que exige ajustes constantes do padrão de movimento do corpo para manutenção da estabilidade. Michael et al. 15 afirmaram que sapatos com formato anatômico permitem a estruturação e função biologicamente normais dos pés. Com uma inspiração no caminhar na areia e na natureza (chamado biomimetismo), existe um calçado comercial para crianças pequenas que fez uso de polímeros para simulação do caminhar e um substrato macio e dinâmico na sua entresola. Com este *design* inspirado na natureza, este sapato se propõe a respeitar a morfologia dos pés da criança (antepé arredondado) e proporcionar melhores condições para o desenvolvimento do pé, principalmente nesta fase de aquisição de marcha. 16

Este estudo investigou se o uso de calçados biomiméticos influencia os padrões de marcha de crianças pequenas. Mais especificamente, este estudo teve como objetivo comparar os parâmetros espaço-temporais e a cinemática da marcha de crianças usando calçados biomiméticos, calçados de uso regular (calçados próprios das crianças, de uso diário) e descalças. A hipótese deste trabalho é que a marcha com calçados biomiméticos teria um padrão mais semelhante ao andar descalço do que o uso de calçados regulares, não biomiméticos.

## Métodos

## Delineamento Experimental e Participantes

Este estudo experimental foi transversal e de medidas repetidas. Vinte crianças com desenvolvimento típico (1 a 3 anos de idade) participaram deste estudo. A amostra foi recrutada com base em amostragem por conveniência com a ajuda de estudantes da universidade e membros da comunidade local. Os critérios de inclusão foram crianças nascidas a termo, de desenvolvimento normal, sem histórico de problemas médicos significativos, capazes de andar de forma independente (ou seja, sem apoio) e tamanho do pé entre 10 e 16,6 cm. Os critérios de exclusão foram crianças maiores de 3 anos, com queixa de dor durante a marcha, em uso de qualquer medicação neurológica sistêmica e incapazes de completar toda a coleta de dados. O termo de consentimento livre e esclarecido foi assinado por todos os pais e os procedimentos foram aprovados pelo Comitê de Ética da Universidade (66806317.1.0000.5149).

## Procedimentos

As configurações do laboratório e os procedimentos experimentais foram adaptados para oferecer um ambiente confortável para as crianças. Antes do registro da marcha, um dos pais segurava a criança pela mão adaptação e exploração do ambiente. Os dados cinemáticos dos membros inferiores foram adquiridos com base em 13 marcadores retrorrefletivos (com 8 mm de diâmetro) colocados no sacro, trocânteres maiores do fêmur, epicôndilos laterais do fêmur, maléolos laterais, tuberosidades calcâneas, primeira e quinta cabeças do metatarso ( Fig. 1 ). A cinemática bilateral dos membros inferiores foi coletada para considerar as assimetrias funcionais descritas em estudos anteriores em crianças saudáveis. 5 Os marcadores laterais dos membros inferiores foram utilizados, já que experimentos-piloto mostraram que os marcadores mediais caem com frequência e precisam ser recolocados durante a avaliação. Os dados foram coletados durante a marcha pelo sistema de análise de movimento Qualisys ProReflex MCU (QUALISYS MEDICAL AB®, Gotemburgo, Suíça) a uma taxa de amostragem de 120 Hz. A ( Fig. 2 ) mostra um fluxograma que ilustra desde a coleta de dados até a análise cinemática. As crianças caminharam em três condições de testes randomizados: descalças, com calçados biomiméticos ( Fig. 3 – Noeh®, www.noeh.com.br , Noeh Baby, Brasil) e com calçados regulares (calçados próprios de uso diário – Fig. 4 ). A condição descalça foi coletada como a avaliação inicial dos parâmetros da marcha para comparação ao uso de calçados. Os calçados biomiméticos (Fig. 3) têm *design* biomimético por apresentar uma entressola dinâmica, imitando o substrato areia, como descrito por Lage (2014). Além disso, nenhuma criança estava familiarizada com esses sapatos biomiméticos antes da coleta de dados. O tamanho dos sapatos foi verificado *a priori* para assegurar o ajuste adequado. Para os calçados regulares ( Fig. 4 ), foi solicitado aos pais que trouxessem os calçados fechados mais confortáveis e de uso diário, à exceção de chinelos ou sandálias abertas. Todas as crianças usaram apenas fraldas descartáveis para aquisição de dados. Cada participante caminhou cerca de dez tentativas em cada condição experimental.

**Fig. 1:** Protocolo de configuração do marcador usado para coleta de parâmetros espaço-temporais e cinemáticos.

**Fig. 2:** Fluxograma mostrando desde a coleta de dados até a análise dos dados: i) a criança foi familiarizada com o ambiente de coleta biomecânica; ii) protocolo de colocação de marcadores passivos em pontos anatômicos; iii) a criança foi orientada a andar em três condições randomizadas (descalça, com sapatos biomiméticos e sapatos comuns); iv) análise biomecânica.

**Fig. 3:** O sapato biomimético. As imagens da esquerda para a direita mostram as vistas de cima, de baixo, do lado medial e lateral do tênis.

**Fig. 4:** Imagem com dois exemplares de sapatos comuns de uso diário.

Durante o registro de dados, as crianças foram encorajadas a caminhar em direção a um dos pais ou pesquisador em velocidade autosselecionada. As crianças andaram sobre uma plataforma elevada (10 cm de altura) e todos os procedimentos foram realizados pelo mesmo examinador experiente e três assistentes científicos treinados. O responsável pela criança e um integrante da equipe de pesquisa permaneceram ao lado dos participantes para evitar quedas.

## Análise de Dados

Os arquivos de dados foram analisados usando o *software* Qualisys Track Manager (QUALISYS MEDICAL AB®, Gotemburgo, Suíça). Os dados cinemáticos foram exportados para processamento no *software* Visual3D (C-Motion, Inc., Rockville, Estados Unidos). Os dados brutos foram inicialmente submetidos a um filtro Butterworth passa-baixa de quarta ordem com corte de 6 Hz. Os seguintes segmentos de membros foram definidos como segmentos rígidos ( Fig. 5 ): pelve (linha entre o marcador do sacro e o marcador do trocânter maior do fêmur), coxa (linha entre o marcador do trocânter maior do fêmur e o marcador do epicôndilo lateral do fêmur), perna (linha entre o epicôndilo lateral do fêmur e o marcador do maléolo lateral) e pé (linha entre o marcador da tuberosidade calcânea e o ponto médio entre a primeira e a quinta cabeças do metatarso). Os eventos necessários para definir o ciclo da marcha foram criados com base no método adaptado para crianças por Ivanenko et al. 17 Em resumo, o ângulo de elevação (α, Fig. 5 ) de cada membro correspondia ao ângulo entre o eixo principal do membro e a vertical (z). O contato inicial e a retirada do pé foram determinados com os maiores e menores valores de ângulo de elevação, respectivamente. Além disso, todos os eventos foram verificados visualmente de forma individual e ajustados quando necessário.

**Fig. 5:** Marcadores anatômicos usados para modelamento dos segmentos corporais dos membros inferiores: pelve, coxa, perna e pé. Os ângulos de elevação (α) foram computados em relação ao eixo principal do membro (do trocânter maior do fêmur à tuberosidade calcânea) e vertical (Z). O contato inicial foi determinado como o maior ângulo de elevação (α) e o *foot-off* foi o menor ângulo de elevação (α).

Trinta a quarenta ciclos de marcha de cada participante foram selecionados para análise. Cada ciclo de marcha foi normalizado para 100% (do contato inicial ao próximo contato inicial). Os seguintes parâmetros espaço-temporais foram extraídos: velocidade da marcha, comprimento e largura da passada. Além disso, o deslocamento vertical médio do centro de massa (CM) do corpo 18 foi calculado com base no marcador retrorrefletivo colocado no sacro. Estudos mostraram que esse marcador estima o deslocamento vertical do CM. 18 Por fim, foram calculadas as seguintes variáveis relacionadas à elevação do pé em relação ao solo: o pico de flexão do joelho 19 20 e a altura máxima do pé durante a fase de balanço. A altura máxima do pé foi considerada o pico máximo de deslocamento vertical do centro de massa do pé.

## Análise Estatística

Os pressupostos de normalidade e homogeneidade de variância foram confirmados antes da execução dos testes inferenciais. Uma análise unicaudal de medidas repetidas (ANOVA) determinou o efeito da condição (descalça, com calçados biomiméticos e com calçados regulares) na velocidade, largura da passada e deslocamento vertical médio de CM. Uma ANOVA bicaudal de medidas repetidas investigou o efeito da condição e do lado (direito e esquerdo) no comprimento da passada, pico de flexão do joelho e altura máxima do pé. Um teste *post-hoc* foi realizado para identificar diferenças pareadas em caso de detecção de um efeito principal significativo à ANOVA. Todas as análises consideraram a probabilidade de erro de tipo I de 0,05. Os tamanhos dos efeitos *f* e *dz* de Cohen foram calculados e interpretados da seguinte maneira: pequenos ( *f* = 0,10 e *dz* = 0,20), médios ( *f* = 0,25 e *dz* = 0,50) e grandes ( *f* = 0,40 e *dz* = 0,80) (Cohen, 1969). A análise estatística foi realizada em IBM SPSS Statistics 22.0 (IBM Corp., Armonk, NY, Estados Unidos).

## Resultados

A Tabela 1 mostra dados descritivos, valores de p, tamanhos de efeito e poder estatístico das análises inferenciais dos parâmetros espaço-temporais e cinemáticos. A princípio, foram recrutadas 20 crianças; no entanto, dados de dezenove crianças (52% de meninas, idade: 14,8 ± 2,0 meses, massa corporal: 10,23 ± 1,19 kg e altura: 0,77 ± 0,04 m) foram analisados. Todas caminhavam sozinhas há mais de 1 mês. Uma criança foi excluída por se recusar a realizar uma das condições analisadas. Além disso, em uma criança, havia dados de ângulo do joelho de apenas um lado com sapatos comuns, já que os dados contralaterais estavam ausentes. Todas as crianças vieram com calçados baixos (sem salto) de uso diário e regular: 33,3% usavam sapatos, conforme ilustrado na Fig. 4 (à direita), e 66,6% usavam tênis, como mostra a Fig. 4 (à esquerda).

**Tabela 1:** Estatísticas descritivas e inferenciais das variáveis espaço-temporais e cinemáticas ( *n* = 19)

**Variável**	**Estatística descritiva – média (DP)**						**ANOVA**			***Post-hoc*** **– Principal efeito da condição**		
	**Sapato biomimético**		**Descalço**		**Sapato comum**		**Efeito principal**		**Efeitos de interação**	**Sapato biomimético x descalço**	**Sapato biomimético x sapato comum**	**Descalço x sapato comum**
	**Direito**	**Esquerdo**	**Direito**	**Esquerdo**	**Direito**	**Esquerdo**	**Condição**	**Lado**				
Parâmetros espaço-temporais												
Velocidade (m/s)	0,59 (0,20)		0,63 (0,21)		0.69 (0.24)		p = 0,05* F (2,36) = 3,21 f = 0,42 Poder = 0,58			p = 0,28 t (18) = -1,12 dz = 0,26 Poder = 0,18	p = 0,02* t (18) = -2,52 dz = 0,58 Poder = 0,66	p = 0,19 t (18) = -1,37 dz = 0,31 Poder = 0,25
Comprimento da passada (m)	0,23 (0,05)	0,21 (0,06)	0,22 (0,05)	0,22 (0,05)	0,24 (0,06)	0,24 (0,06)	p = 0,04* F (2,36) = 3,58 f = 0,45 Poder = 0,63	p = 0,35 F (1,18) = 0,92 f = 0,23 Poder = 0,15	p = 0,06 F (2,36) = 3,08 f = 0,42 Poder = 0,56	p = 0,96 t (18) = -0,06 dz = 0,01 Poder = 0,05	p = 0,02* t (18) = -2,49 dz = 0,57 Poder = 0,65	p = 0,06 t (18) = -1,98 dz = 0,45 Poder = 0,47
Largura da passada (m)	0,11 (0,03)		0,11 (0,02)		0,11 (0,02)		p = 0,71 F (2,36) = 0,34 f = 0,14 Poder = 0,10					
Parâmetros cinemáticos												
Deslocamento médio vertical de CM (m)	0,016 (0,006)		0,016 (0,006)		0,019 (0,006)		p = 0,02* F (2,36) = 4,13 f = 0,48 Poder = 0,69			p = 0,82 t (18) = -0,23 dz = 0,05 Poder = 0,06	p = 0,01* t (18) = -2,99 dz = 0,68 Poder = 0,81	p = 0,05* t (18) = -2,06 dz = 0,47 Poder = 0,50
Pico de flexão do joelho ( ^o^ )	76,5 (9,4)	71,1 (9,0)	71,0 (11,0)	67,5 (9,2)	75,0 (9,1)	75,1 (13,8)	p = 0,01* F (1,5,34) = 6,48 f = 0,62 Poder = 0,80	p = 0,06 F (1,17) = 3,92 f = 0,48 Poder = 0,46	p = 0,19 F (1,3,34) = 1,73 f = 0,31 Poder = 0,27	p < 0,01* t (18) = 3,92 dz = 0,90 Poder = 0,96	p = 0,48 t (17) = -0,73 dz = 0,17 Poder = 0,10	p = 0,01* t (17) = -2,81 dz = 0,66 Poder = 0,73
Altura máxima do pé (m)	0,098 (0,017)	0,096 (0,017)	0,089 (0,014)	0,089 (0,013)	0,093 (0,019)	0,092 (0,018)	p = 0,07 F (2,36) = 2,85 f = 0,40 Poder = 0,52	p = 0,41 F (1,18) = 0,72 f = 0,20 Poder = 0,13	p = 0,87 F (2,36) = 0,15 f = 0,10 Poder = 0,07			

**Observação:** DP = Desvio-padrão; CM = centro de massa, ^*^ = *p* < 0,05; *F* (x,y) = F estatístico (graus de liberdade), *f* = tamanho do efeito do f de Cohen, *dz* = = tamanho do efeito do *dz* de Cohen.

## Parâmetros Espaço-temporais

A velocidade diferiu nas três condições ( **Tabela 1** ). A análise *post-hoc* mostrou que as crianças andavam mais devagar quando usavam calçados biomiméticos do que calçados regulares. Não houve diferenças em relação aos pés descalços.

O comprimento da passada também foi diferente entre as condições (Tabela 1). A análise *post-hoc* revelou que as crianças apresentaram comprimento menor da passada ao usarem calçados biomiméticos em comparação a calçados regulares. Não houve diferença no comprimento da passada entre as condições calçada e descalça. Além disso, não houve diferença entre comprimentos da passada direita e esquerda nem efeito de interação entre condição e lado.

A largura da passada não foi diferente entre as condições (Tabela 1).

## Parâmetros Cinemáticos

O deslocamento vertical médio do CM foi maior em crianças com calçados regulares em comparação àquelas descalças ou com calçados biomiméticos (Tabela 1). Além disso, esta variável não apresentou diferença estatística entre as condições com calçados biomiméticos e descalço.

O pico de flexão do joelho também foi diferente entre as condições. A análise *post-hoc* mostrou que as crianças com o uso de sapatos apresentaram maior pico de flexão do joelho do que com os pés descalços. Esta variável não foi diferente entre as condições com calçados. Além disso, não houve diferença entre os picos de flexão do joelho direito e esquerdo nem efeito de interação entre a condição e o lado.

A altura máxima do pé durante a fase de balanço não foi diferente entre as condições ou lados e não houve interação entre condição e lado.

## Discussão

O principal achado deste estudo foi que os calçados com *design* biomimético têm impacto menor do que os calçados comuns nos parâmetros espaço-temporais e na cinemática da marcha de crianças saudáveis. Este estudo comparou as características da marcha de crianças pequenas em três condições: calçados biomiméticos, calçados regulares de uso diário e pés descalços. Há poucas evidências de qualidade e de confiabilidade razoável que afirmam que calçados, principalmente os infantis, alterariam os parâmetros biomecânicos da marcha. 20 21 Este trabalho indicou que calçados com *design* biomimético têm um impacto menor nos padrões de marcha de crianças pequenas, sendo similares ao andar descalço.

Como medida global da marcha, os parâmetros espaço-temporais podem fornecer informações sobre as diferenças entre as três condições de caminhada. A velocidade de marcha o comprimento da passada apresentaram diferenças significativas, com grande tamanho de efeito, entre as condições. Estudos anteriores relataram que andar calçado aumenta a velocidade da marcha em comparação aos pés descalços. 2 22 Por outro lado, outros autores não observaram esta diferença. 3 7 20 23 No presente estudo, a velocidade da marcha com calçados biomiméticos não foi diferente em comparação aos pés descalços; no entanto, houve diferenças entre os calçados biomiméticos e regulares. A entressola biomimética forma uma superfície de contato irregular para a região plantar do pé. Esse desafio pode ter contribuído para a redução da velocidade de marcha das crianças. Shkuratova et al. 24 descreveram que a menor velocidade de marcha pode melhorar sua estabilidade. Quando a velocidade de marcha foi diretamente manipulada em adultos jovens, as velocidades menores diminuíram a instabilidade dinâmica local, apesar do aumento da variabilidade. 24 Assim, a redução da velocidade pode ser uma adaptação da criança para melhorar a estabilidade da marcha ao usar sapatos não usuais que proporcionam uma superfície de contato mais macia para os pés.

O comprimento da passada foi maior com calçados regulares em comparação aos calçados biomiméticos. O aumento do comprimento da passada com o uso do calçado corroboa com a literatura prévia. 2 10 20 22 23 No entanto, usar calçados biomiméticos não foi diferente de estar descalço. Além disso, o deslocamento vertical médio do CM também não foi afetado pelos calçados biomiméticos em comparação aos pés descalços. O movimento do CM durante a marcha pode representar um indicador resumido do movimento de todo o sistema corporal. 25 26 Esse comportamento pode fornecer informações gerais sobre a mecânica da marcha quanto ao gasto energético e a eficiência mecânica. Nossos resultados indicaram que o deslocamento vertical médio do CM com calçados regulares foi maior do que nas outras duas condições. Este resultado pode estar relacionado com a maior velocidade 26 27 obtida com o uso de calçados regulares, de uso diário, também apresentada em nossos dados. Além disso, essa diferença de comportamento do CM pode contribuir para um maior custo metabólico da marcha 28 com calçados regulares em comparação a calçados biomiméticos ou pés descalços.

O pico de flexão do joelho diferiu entre as condições com calçados e pés descalços (tamanho de efeito grande). Nossos resultados condizem com a literatura 2 já que um pequeno aumento no pico de flexão do joelho foi observado com o uso de calçados em comparação aos pés descalços. Esta alteração pode ser atribuída ao aumento de peso relacionado ao uso dos calçados. O maior do peso dos calçados distalmente pode aumentar a flexão do joelho durante a marcha. Dominici et al. 29 concluíram que a maior elevação do pé poderia ser uma estratégia simples e segura para evitar possíveis tropeços e quedas e reduzir o efeito do arrasto involuntário do pé e a atividade de dorsiflexão.

Até onde sabemos, nenhum estudo investigou o impacto de calçados com *design* biomimético em crianças. O presente estudo não rastreou os segmentos corporais de forma tridimensional, o que limitou os resultados. O rastreamento tridimensional requer a colocação de mais marcadores retrorreflexivos nas crianças. Durante o estudo-piloto, descobrimos que algumas crianças não toleram a colocação de múltiplos marcadores em seus corpos. Além disso, os marcadores anatômicos utilizados para definir o segmento do pé foram colocados sobre os sapatos e não diretamente na pele do pé. Outra limitação foi que os participantes usavam calçados regulares diferentes. Uma vez que escolheram o mais habitual e confortável, as características dos calçados regulares diferiram entre todos os participantes. Além disso, os resultados deste estudo foram de efeito imediato do uso do calçado biomimético. Assim, nossa pesquisa não considerou o impacto dos calçados biomiméticos em longo prazo. Mais pesquisas sobre o uso de calçados com *design* biomimético por períodos maiores podem melhorar a compreensão do efeito desses calçados.

## Conclusão

O presente estudo mostrou que o uso de calçados biomiméticos não alterou a velocidade de caminhada, o comprimento da passada, a largura da passada e o deslocamento vertical do CM em comparação à marcha descalça em crianças pequenas durante o início da fase de aquisição da marcha. O pico de flexão do joelho foi influenciado pelo uso de ambos os calçados avaliados. Os calçados regulares geraram maior deslocamento vertical do CM do que os calçados biomiméticos e a marcha descalça. Portanto, o *design* do calçado biomimético pode proporcionar uma experiência de marcha semelhante ao andar descalço, com menor impacto no padrão de caminhada.
